# Adjuvant Therapy Using Mistletoe Containing Drugs Boosts the T-Cell-Mediated Killing of Glioma Cells and Prolongs the Survival of Glioma Bearing Mice

**DOI:** 10.1155/2018/3928572

**Published:** 2018-08-27

**Authors:** Sonja Schötterl, Stephan M. Huber, Hans Lentzen, Michel Mittelbronn, Ulrike Naumann

**Affiliations:** ^1^Molecular Neuro-Oncology, Hertie Institute for Clinical Brain Research and Center Neurology, University of Tübingen, Otfried-Müller-Str. 27, 72076 Tübingen, Germany; ^2^Department of Radiation Oncology, University of Tübingen, Hoppe-Seyler-Str. 3, 72076 Tübingen, Germany; ^3^MELEMA Pharma GmbH, Hamburg, Germany; ^4^Luxembourg Centre of Neuropathology (LCNP), Luxembourg, 1 Rue Louis Rech, L-3555 Dudelange, Luxembourg; ^5^NORLUX Neuro-Oncology Laboratory, Luxembourg Institute of Health (LIH), Luxembourg, 84 Rue Val Fleuri, L-1526 Luxembourg, Luxembourg; ^6^Neurological Institute (Edinger Institute), Goethe University Frankfurt, Heinrich-Hoffmann-Str. 7, 60528 Frankfurt/Main, Germany; ^7^Laboratoire Nationale de Santé, Dudelange (LNS), 1 Rue Louis Rech, L-3555 Dudelange, Luxembourg; ^8^Luxembourg Centre for Systems Biomedicine (LCSB), University of Luxembourg, 7 Avenue des Hauts-Fourneaux, 4362 Esch-sur-Alzette, Luxembourg

## Abstract

*Viscum album L.* extracts (VE) are applied as complementary cancer therapeutics for more than one century. Extracts contain several compounds like mistletoe lectins (ML) 1-3 and viscotoxins, but also several minor ingredients. Since ML-1 has been described as one of the main active components harboring antitumor activity, purified native or recombinant ML-1 has been also used in clinical trials in the last years. The present study examined and compared the immunoboosting effects of three ML-1 containing drugs (the extract ISCADOR Qu, the recombinant ML-1 Aviscumine, and purified native ML-1) in the context of the T-cell mediated killing of glioma cells. Additionally we examined the possible underlying T-cell stimulating mechanisms. Using cocultures of immune and glioma cells, a PCR-based microarray, quantitative RT-PCR, and an antibody-based array to measure cytokines in blood serum, immunosupporting effects were determined. A highly aggressive, orthotopic, immunocompetent syngeneic mouse glioma model was used to determine the survival of mice treated with ISCADOR Qu alone or in combination with tumor irradiation and temozolomide (TMZ). Treatment of glioblastoma (GBM) cells with ISCADOR Qu that contains a high ML concentration, but also viscotoxins and other compounds, as well as with Aviscumine or native ML-1, enhanced the expansion of cancer cell-specific T-cells as well as T-cell-mediated tumor cell lysis, but to a different degree. In GBM cells all three ML-1-containing preparations modulated the expression of immune response associated genes.* In vivo,* subcutaneous ISCADOR Qu injections at increasing concentration induced cytokine release in immunocompetent VM/Dk-mice. Finally, ISCADOR Qu, if applied in combination with tumor irradiation and TMZ, further prolonged the survival of glioma mice. Our findings indicate that ML-1 containing drugs enhance anti-GBM immune responses and work in synergy with radiochemotherapy. Therefore, adjuvant mistletoe therapy should be considered as an auspicious treatment option for glioma patients.

## 1. Introduction

GBM is the most common primary brain tumor in adults. Even at best care, optimal surgical resection of the tumor followed by irradiation and chemotherapy, the median overall survival does not exceed 1.5 years [1]. This is mainly based on the malignant characteristics of GBM. GBM grow infiltratively into the healthy brain making a complete resection often impossible and show a strong vascularization and multidrug resistance [2]. Additionally, GBM is one of the most immunosuppressive cancers. GBM cells escape natural killer (NK) cells by downregulation of NKG2D ligands. Downregulation of MHC molecules as well as secretion of immunosuppressive cytokines by GBM cells blocks T-cell activation and pushes the development of immunosuppressive regulatory T-cells. Additionally, GBM cells show enhanced expression of T-cell exhaustion ligands (for review see [3]).

Extracts from the semiparasitic plant* Viscum album L.* (VE) are used as adjuvant cancer therapeutics. The compositions of these extracts differ in dependence on the host tree the plant is growing on, due to different extraction methods and the harvest season. Anticancer effects of VEs are primarily attributed to mistletoe lectins (MLs). In particular, ML-1 provides anticancer activity [4]. Further ingredients of VE are viscotoxins (VT), triterpenes, flavonoids, phytosterols, and oligo- and polysaccharides that provide anticancer activity themselves or that potentiate ML effects [5–7]. Nowadays, purified or recombinant ML-1 is also used for cancer therapy [8, 9]. MLs are ribosomal inhibitor type 2 proteins (RIP) and contain two subunits, the cytotoxic *α*-chain and the *β*-chain. The latter mediates the specific binding to its receptor CD75s [10, 11]. CD75s is expressed on immune cells but is upregulated on several cancer cells [12–14], also on glioma cells. ML containing drugs have been identified to attenuate tumor cell motility, to change the expression of cancer-associated genes, to induce cell death, to reduce tumor growth, and to boost anticancer immune responses [4, 15–17]. We have previously shown that the ML-rich extract ISCADOR Qu, as well as Aviscumine, a recombinant ML-1, but not the ML-poor extract ISCADOR P, promote NK-cell activity against glioma cells [15, 18]. In clinical trials ISCADOR also demonstrated a positive effect on NK-cell functions [19]. Beyond this, mistletoe extracts trigger monocyte-derived macrophage activity, dendritic cell (DC) maturation, and induce a specific T-helper cell reaction [20, 21]. Additionally, they neutralize tumor-induced immunosuppression, at least* in vitro* [22].* In vivo* both, extracts and purified MLs, increased the number of leucocytes and granulocytes and enhanced the blood level of granulocyte-macrophage colony stimulating factor (GM-CSF), interferon (IFN)-*γ*, and interleukin (IL)-5 [23]. ML-mediated enhanced IL-1*β*, -6, -10 and tumor necrosis factor (TNF)-*α* expression has been described in immune cells, even if quantitative differences in the immunomodulatory effects of the different ML preparations have been observed [24]. Combined these findings suggest that ML-1 containing drugs might be beneficial to support antitumoral immune responses also in a highly immunosuppressive tumor like GBM. We tested this hypothesis with a particular emphasis on the activation of T-cells and compared the effects of three different ML-1-containing preparations: ISCADOR Qu is a ML-rich, fermented extract generated from mistletoe plants growing on oak trees. Aviscumine is a nonglycosylated, recombinant ML-1 and native ML-1 was purified from ash tree mistletoes. We demonstrate that all three preparations enhanced the expansion and anti-glioma cell activity of T-cells to a different extent, probably by differentially modulating the expression of immune response related genes in the tumor cells. Repeated ISCADOR Qu injections alone, or even better if administered in combination with tumor irradiation and chemotherapy, prolonged the median survival of glioma bearing mice.

## 2. Materials and Methods

### 2.1. ML Containing Preparations

ISCADOR Qu was provided by the ISCADOR AG (Lörrach, Germany). ML and VT contents were ISCADOR Qu_20_ (Charge 4080/3: 20 mg/ml of extract, i.e., ML 1095 ng/ml, VT 48 *μ*g/ml). Aviscumine, a GMP quality recombinant ML-1 produced in* E. coli,* was provided from MELEMA Pharma GmbH (Hamburg Germany) and purified native ML-1 isolated from ash tree mistletoes by Abnoba GmbH (Pforzheim, Germany).

### 2.2. Cell Culture

LNT-229 glioma cells (N. de Tribolet, Lausanne, Switzerland) were maintained in Dulbecco's modified Eagle's medium (DMEM; GIBCO Life Technologies, Eggenstein, Germany) containing 10% fetal calf serum (GIBCO Life Technologies, Eggenstein, Germany), penicillin (100 U/ml), and streptomycin (100 *μ*g/ml) (P/S). Peripheral blood mononuclear cells (PBMC) were isolated from blood of healthy donors [15] and were maintained in RPMI1640 (2 mM glutamine 5% human serum, penicillin, and streptomycin (all from Sigma-Aldrich, Taufkirchen, Germany)). The cells were regularly tested to be free of mycoplasma using the MycoAlert mycoplasm detection kit (Lonza, Rockland, ME, USA). Cell viability was measured using the MTT assay (Abcam, Cambridge, UK).

### 2.3. Microarray Analysis and Quantitative RT-PCR

The TaqMan human immune response expression microarray (Thermo Fisher Scientific, Waltham, MA, USA) and quantitative RT-PCR (GoTaq-PCR Master, Promega, Madison, WI, USA) were performed as previously described [16] using the following primers: GAPDH-frwd TGCACCACCAACTGCTTAGC, GAPDH-rev GGCATGGACTGTGGTCATGAG; IL-6-frwd TTCCTGCAGAAAAAGGCAAAGA, IL-6-rev AAAGCTGCGCAGAATGAGATG; IL-8-frwd GTGGAGAAGTTTTTGAAGAGGGC, IL-8-rev CACTTCATGTATTGTGTGGGTCT; IL-10-frwd CTTGATGTCTGGGTCTTGGTT, IL-10-rev GCTGG-AGGACTTTAAGGGTTA; IL-12A-frwd CCAGAAGGCCAGACAAACTCTA, IL-12A-rev GCCAGGCAACTCCCATTAGTT; TNF-frwd CACAGTGAAGTGCTGGCAAC, TNF-rev AGGAAGGCCTAAGGTCCACT; BCL2-frwd GGTGAACTGGGGGAGGATTG, BCL2-rev GCCCAGACTCACATCACCAA; HMOX1-frwd CAGGCTCCGCTTCTCCGATG, HMOX1-rev GGAGCCAGCATGCCTGCATTC; SELE-frwd GCCTGCAATGTGGTTGAGTG, SELE-rev GGTACACTGAAGGCTCTGGG.

### 2.4. Expansion and Activation of T-Cells

PBMCs were isolated from the peripheral blood of human healthy, anonymized volunteers by density gradient centrifugation using Biocoll Separating Solution (Biochrom/Merck, Berlin, Germany) and were cocultured with irradiated (30 Gy) human LNT-229 glioma cells that were pretreated (or left untreated) for 24 h with ISCADOR Qu, Aviscumine, or native ML-1 (8 ng/ml of ML). T-cell expansion was performed as described [25]. T-cells were purified using the T-cell isolation kit (Miltenyi, Bergisch Gladbach, Germany). T-cell purity and activity were determined by flow cytometry (FACS) using anti-human CD3, CD4, CD8, CD69, and CD56 antibodies (Biolegend, Fell, Germany). On average > 95% of cells were CD3^+^, and in this population > 50% of cells were CD69^+^.

### 2.5. Flow Cytometry

T-cells were washed with FACS buffer (0.5% BSA, 2 mM EDTA, 0.02% NaN_3_, PBS), blocked with 0.5% Gamunex (Grifols, Barcelona, Spain), and stained with propidium iodide (PI), Annexin V, anti-human CD3, CD25, HLA-DR, CD4, CD8, CD56, or CD69 (all from Biolegend, London, UK). The cells were washed twice and analyzed using a CyAn ADP flow cytometer (Beckman Coulter, Krefeld, Germany). Quantification was done using FloJo Software (FloJo, LLC, Ashland, Oregon, USA).

### 2.6. T-Cell Cytotoxicity Assay

T-cell cytotoxicity was measured as described [15]. LNT-229-Luciferase expressing cells (LNT-229-Luc) were left untreated or were treated for 24 h with ISCADOR Qu, Aviscumine, or native ML-1 (8 ng/ml of ML). The cells were washed intensively to remove residual MLs, the medium was changed to RPMI1640 and T-cells were added for 4 h at different effector to target (E:T) ratios (0.5 : 1, 1 : 1, 5 : 1, 10 : 1, 20 : 1). Sodium dodecyl sulfate (SDS, 1%) was used as a control for complete cell lysis. Luciferase activity was measured and used to calculate cell lysis. To exclude cytotoxic or growth inhibitory effects induced by the ML containing drugs, values derived by the T-cell toxicity assay were normalized to the cell density of glioma cells that were grown in parallel at same conditions, but in the absence of T-cells.

### 2.7. Animal Experiments and Cytokine Detection in Mouse Blood Serum

The ISCADOR Qu-mediated cytokine induction was measured in 3-6-month-old ISCADOR Qu or PBS injected (s.c.) VM/Dk mice. Mice were injected thrice a week at days 2, 4, and 7 for three weeks (100 *μ*l: 2 x 0.8 ng/ml, 2 x 8 ng/ml, 4 x 80 ng/ml; n=4). This application schema was chosen to mimic the application schema that is used to treat cancer patients [26]. The mice were sacrificed, and blood serum was collected and used for the mouse inflammation antibody array (G-series, AAM-INF-G1-8, RayBiotech, Norcross, GA, USA) according to the manufacturer's protocol. For induction of glioma, 5000 murine SMA-560 glioma cells were implanted into the striatum of 3-6-month-old VM/Dk mice [27]. Seven days later the mice were randomly split into treatment groups. Tumors were locally irradiated (3 Gy, 6 MV photons) at day 7 after tumor cell implantation at a dose rate of 4 Gy/min using a linear accelerator (LINAC SL25 Philips). Chemotherapy was applied by intraperitoneal injections of temozolomide (TMZ, 2.5 mg/kg) at days 8, 14, and 20. Adjuvant mistletoe therapy was performed by s.c. injections of ISCADOR Qu from day 8 onwards (thrice a week as described above; 100 *μ*l; 2 x 0.8 ng/ml, 2 x 8 ng/ml, 2 x 80 ng/ml,). As a control, cohorts were treated with PBS instead of ISCADOR Qu and did not receive tumor irradiation or TMZ injections, respectively. The mice were sacrificed upon the onset of tumor-related symptoms.

### 2.8. Statistical Analysis

Unless stated otherwise, figures show the mean of at least three independent experiments ± standard deviation (SD).* In vitro* data (n ≥ 5) were tested for normal distribution and passed the Kolmogorov-Smirnov normality test for normal distribution using Instat (GraphPad, San Diego, USA). Differences were tested for significance by the unpaired Student's t-test (^*∗*^ *p* < 0.05; ^*∗∗*^ *p* < 0.01; ^*∗∗∗*^ *p* <0.001) using Excel (Redmond, WA, USA). Kaplan-Meier survival curves were generated and tested for significance using the log-rank test (^*∗*^ *p* < 0.05; ^*∗∗*^ *p* < 0.01; ^*∗∗∗*^ *p* < 0.001) by applying JMP 13 software (SAS, Böblingen, Germany). Probability (p) values were adjusted for multiple comparisons by Bonferroni correction.

## 3. Results

### 3.1. ML-1 Containing Drugs Support the Expansion of Cancer-Specific T-Cells and the T-Cell-Mediated Killing of Glioma Cells

**Figure 1:**
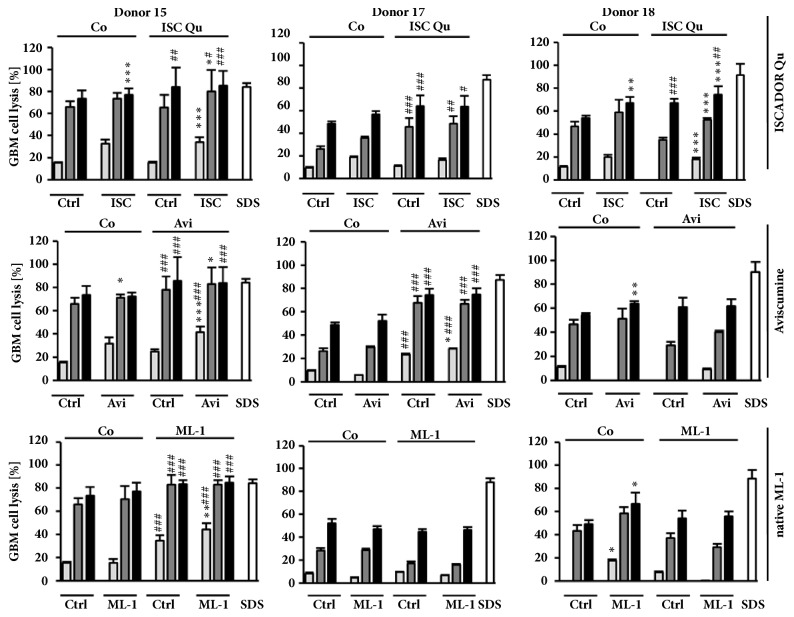
**T-cell-mediated killing of glioma cells is supported by ML-1 containing drugs**. Results for three individual PBMC donors (# 15, 17, 18) are depicted. PBMCs were cocultured with untreated (Co, label on top of bars) or ISCADOR Qu (ISC), Aviscumine (Avi), or native ML-1 (ML-1) pretreated LNT-229 cells for 3-5 weeks to achieve allogenic T-cell expansion and activation. Activated T-cells were added in different effector to target (E:T) ratios (5 : 1, light grey bars; 10 : 1, dark grey bars; 20 : 1, black bars) to untreated (Ctrl, x-axis) or pretreated LNT-229-Luc-cells for 4 h (Ctrl: ISC, Avi, ML-1; 8 ng/ml of ML; shown on the x-axis). Luciferase activity was measured and used to calculate the amount of glioma cell lysis (mean ± SD of 6-8 replicates; Student's t-test compared to Ctrl (*∗*) or Co (#) ^*∗*/#^P < 0.05, ^*∗∗*/##^P < 0.01, ^*∗∗∗*/###^P < 0.001 after Bonferroni correction for n pair comparisons).

T-cells are potent warriors against cancer. However, glioma cells develop several mechanisms to escape T-cell attacks. We were interested whether the extract ISCADOR Qu or pure native or recombinant ML-1 helps to overcome immunosuppressive effects of GBM cells and whether these agents support the T-cell-mediated killing of glioma cells. We treated LNT-229-Luc cells for 24 h with a subtoxic concentration of ISCADOR Qu, Aviscumine, or native ML-1 (8 ng/ml, [16]). After removal of residual components by intensive washing steps, glioma cells were cocultured with activated T-cells as described in the Methods part. The increase of glioma cell lysis was variable and dependent on the T-cell donor; however there was a slight, but significant enhancement of glioma cell lysis by 2/3 donors if glioma cells were pretreated with ISCADOR Qu or Aviscumine whilst native ML-1 showed this effect for only one donor (Figure 1, 1^st^ to 6^th^ bar of each graph).

We were also interested whether ISCADOR Qu, Aviscumine, or native ML-1 helps to expand cancer-specific T-cells. To this end, PBMCs isolated from healthy human donors were cocultured during the priming/expansion phase with ISCADOR Qu, Aviscumine, or native ML-1-pretreated glioma cells. T-cells generated this way showed more glioma lysis activity than T-cells generated by coculturing with nontreated glioma cells (Figure 1, 7^th^ to 12^th^ bar of each graph). Even LNT-229-Luc glioma cells which have never been treated with ML-1 containing preparations were killed more efficiently by the population of T-cells generated by this approach. In the context of generating cancer-specific activated T-cells, native ML-1 showed only minor effects and only in T-cells of one donor (donor 15). Aviscumine showed a supporting effect in 2/3 T-cell-donors (donors 15 and 17) whilst ISCADOR Qu significantly facilitated the priming of cancer-specific T-cells from PBMCs of all three donors.

### 3.2. ISCADOR Qu or Pure ML-1 Preparations Modulate the Expression of Immune Response Related Genes

**Figure 2:**
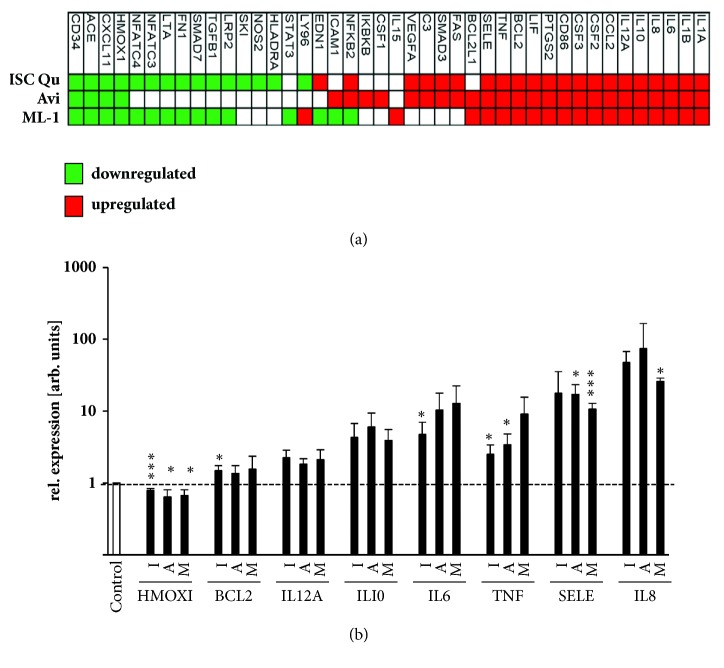
**ML-1 containing drugs modulate the expression of inflammation associated genes**. (a) Heatmap showing changes in the expression of immune response-associated genes in LNT-229 cells treated for 24 h with either ISCADOR Qu (ISC Qu), Aviscumine (Avi), or native ML-1 (ML-1; each 8 ng/m of ML) as determined by microarray analysis. (b) qRT-PCR analysis of genes that were found to be differentially expressed in treated LNT-229 cells (8 ng/ml; 24 h) as indicated in (a). GAPDH serves as reference (I: ISCADOR Qu, A: Aviscumine, M: native ML-1; means ± SD, n=3; Student's t-test compared to control; ^*∗*^P < 0.05, ^*∗∗*^P < 0.01, ^*∗∗∗*^P < 0.001 after Bonferroni correction for n pair comparisons).

MLs provide effects not only by their function as RIPs, but also by changes in gene expression [16]. To analyze whether ISCADOR Qu, Aviscumine, or native ML-1 alters the expression of immunomodulating genes, LNT-229 cells were treated for 24 h with ISCADOR Qu, Aviscumine, or native ML-1 at a concentration of 8 ng/ml of ML which induces lesser than 15% of cell number reduction and is far below the calculated IC_50_ [15, 16]. To analyze changes in gene expression, a PCR-based microarray, followed by qPCR validation, was used (Figure 2). mRNAs presented on the microarray harboring immune functions fall into nine classes: transcription factors, cell surface receptors, stress response genes, proteases and oxireductases, and proteins involved in signal transduction as well as cytokines, chemokines, and their receptors. Additionally, protein kinases and cell cycle proteins are also presented on the microarray. In the panel of 92 immune-related genes, 42 mRNAs were found to be differentially expressed. 15 mRNAs were down- and 21 upregulated by ISCADOR Qu whilst only 4 mRNAs were down- and 24 upregulated by Aviscumine. Native ML-1 induced 15 and repressed 19 mRNAs. Upregulated mRNAs include mainly proinflammatory factors like interleukin (IL)-1A, IL-1B, IL-6, IL-8, IL-12A, IL-15, inhibitor of nuclear factor kappa B kinase subunit beta (IKBKB), C-C motif chemokine ligand (CCL)-2, colony stimulating factor (CSF)-1, -2 and -3, CD86, prostaglandin-endoperoxide synthase (PTGS)2/ COX-2, tumor necrosis factor (TNF), E-selectin/CD62 (SELE), complement component 3 (C3), and vascular endothelial growth factor A (VEGFA). Besides proinflammatory factors, the antiapoptotic protein BCL2-like-1 (BCL2L1) was upregulated upon ML treatment. In the panel of downregulated mRNAs, members of the (TGF)-*β* pathway like TGF-*β* and SMAD3 were identified. The expression of the anti-inflammatory heme oxygenase 1 gene (HMOX1) and of inflammatory-associated angiotensin converting enzymes (ACE) was also reduced. However, the cohort of downregulated genes also contains some proinflammatory factors like CXCL11. In summary, treatment of glioma cells with ISCADOR Qu, Aviscumine, or native ML-1 enhanced the expression of genes that facilitate a more inflammatory phenotype.

**Figure 3:**
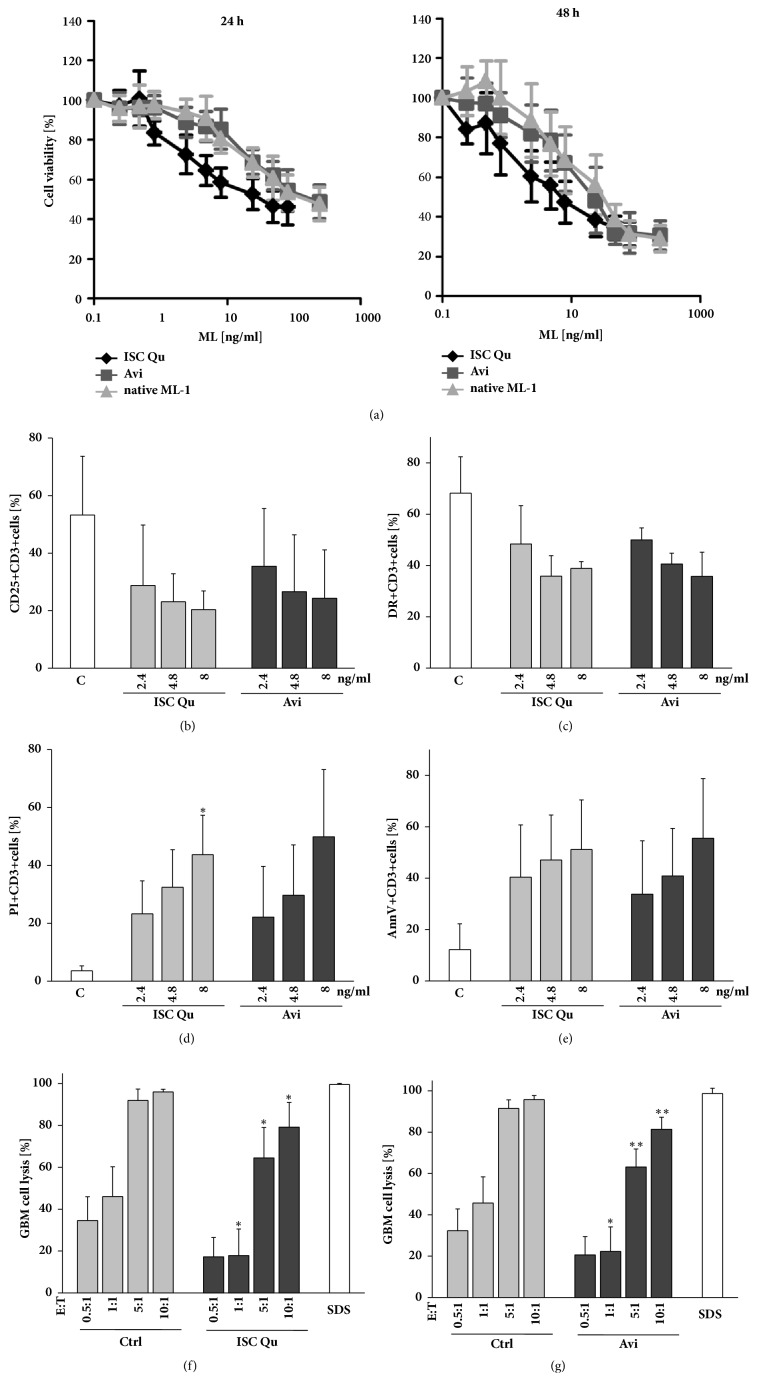
**ML-1 containing drugs induce cell death in PBMCs and reduce their lytic activity**. PBMCs were treated with ISCADOR Qu (ISC Qu), Aviscumine (Avi), or native ML-1 for 24 h or 48 h. (a) Cell viability of PBMCs was measured using MTT. (b–e) FACS analysis of activated T-cells treated for 48 h with ISCADOR Qu (ISC Qu) or Aviscumine (Avi). CD25 (b) and HLA-DR (c) surface expression. Cell death was measured by PI staining (d) and breakdown of the phospholipid asymmetry of the plasma membrane by AnnexinV binding (e). (f-g) Glioma cell lysis: Activated T-cells were left untreated (Ctrl) or were treated for 24 h with 2.4 ng/ml ISCADOR Qu (f) or Aviscumine (g). Afterwards these cells were cocultured for 4 h at different effector to target (E:T) ratios with LNT-229-Luc-cells. The remaining luciferase activity was measured and used to calculate the amount of glioma cell lysis (means ± SD, n=3; Student's t-test compared to control ^*∗*^P < 0.05, ^*∗∗*^P < 0.01, ^*∗∗∗*^P < 0.001 after Bonferroni correction for n pair comparisons).

Since in cancer patients extracts like ISCADOR Qu or pure ML-1 preparations like Aviscumine are mainly applied as s.c. injections, we examined whether there are direct effects of these drugs on the viability of immune cells. We treated PBMCs with increasing concentrations of ISCADOR Qu, Aviscumine, or native ML-1 and calculated IC_50_ values. Whereas in LNT-229 cells the IC_50_ concentration is 37 ng/ml (ISCADOR Qu), 95 ng/ml (Aviscumine), and >240 ng/ml (ML-1), respectively [16], PBMCs were more sensitive towards ISCADOR Qu (26 ng/ml) and native ML-1 (144.2 ng/ml) but slightly more resistant to Aviscumine (171 ng/ml; Figure 3(a)). Nevertheless, treatment of PBMCs with ISCADOR Qu or Aviscumine even at a concentration of 2.4 ng/ml of ML (which is far below the 24 h IC_50_ values we calculated for these cells) reduced the expression of CD25 (Figure 3(b)) and HLA-DR (Figure 3(c)) on CD3^+^ cells, induced cell death in PBMCs (Figures 3(d) and 3(e)), and reduced the immune-cell mediated glioma cell lysis (Figures 3(f) and 3(g)).

### 3.3. Adjuvant ISCADOR Qu Application Prolonged the Survival of Glioma Mice

Therapeutic effects of an adjuvant mistletoe therapy were determined in the highly aggressive, TMZ-resistant, immunocompetent SMA-560/VM/Dk mouse glioma model. We have chosen ISCADOR Qu for our* in vivo* studies since our* in vitro* data demonstrated that, among the three tested ML-1-containing preparations, ISCADOR Qu provided superior immunosupporting effects compared to Aviscumine and native ML-1, especially in the generation and expansion of tumor-specific T-cell (Figure 1).

**Figure 4:**
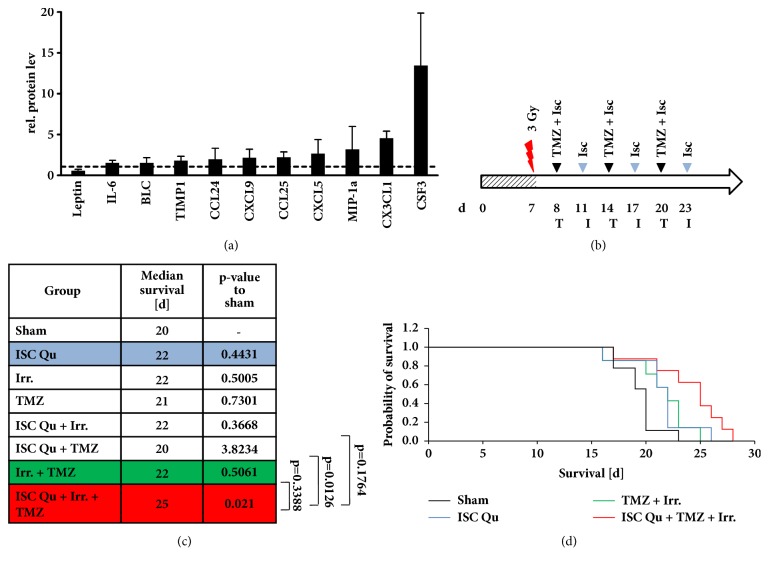
**Adjuvant ISCADOR Qu therapy prolonged the survival of glioma bearing mice**. (a) Differential expression of cytokines in the serum of VM/DK mice injected with ISCADOR Qu or PBS as a control as described in the Methods part (means ± SD, n=4 animals). (b) Treatment schema of SMA-560 glioma bearing VM/Dk mice: Day 0 (d_0_) depicts tumor cell implantation, irradiation (flash), ISCADOR Qu (I), ISCADOR Qu + TMZ (T). (c) Median survival of treatment groups (n=7 to 8 animals per group; p-values were defined using the log-rank test after Bonferroni correction for n pair comparisons). (d) Kaplan Mayer survival curves of the sham, ISCADOR Qu, TMZ + irradiation, and combined treatment (ISC Qu + TMZ + irradiation) groups.

To analyze ISCADOR Qu-mediated immune stimulation we repeatedly injected VM/Dk mice for three weeks with increasing doses of ISCADOR Qu as described in the Methods part. This application schema was used to mimic the protocol that is commonly used for cancer patients. At the end of the treatment period we measured serum levels of 40 inflammatory and immunomodulating cytokines. In ISCADOR Qu treated mice, 11 proinflammatory proteins were upregulated: IL-6, B lymphocyte chemoattractant (BLC), tissue inhibitor of metalloproteinase (TIMP)-1, chemokine ligand (CCL)-24, CCL25, chemokine (C-X-C motif) ligand (CXCL)-9, CXCL5, macrophage inflammatory protein (MIP)-1*α*, chemokine (C-X3-C motif) ligand (CX3CL)-1, and colony stimulating factor (CSF)-3. Only the autoimmune disease associated adipokine leptin was downregulated (Figure 4(a)).

To analyze therapeutic effects of adjuvant mistletoe therapy, VM/Dk glioma mice were tumor irradiated and/or TMZ-treated. Some groups were additionally subjected to ISCDAOR Qu injections (for treatment pattern and groups see Figures 4(b) and 4(c) and Methods). As a control, mice were injected with ISCADOR Qu alone or were solely subjected to tumor irradiation and/or TMZ. No single or double treatment led to a significant prolongation of the median survival (Figure 4(c)). In the cohort of ISCADOR Qu treated mice a slight extension of survival time was detectable which was comparable to that achieved by irradiation plus TMZ, indicating that repeated s.c. injections of ISCADOR Qu showed therapeutic effects. The combination of all treatment options (ISCADOR Qu + irradiation + TMZ), even if not significant in comparison to standard treatment (irradiation + TMZ), further prolonged the survival time. Nevertheless, adjuvant ISCADOR Qu application significantly prolonged the survival of VM/Dk glioma mice if compared to control treated animals (p=0.021) whilst standard glioma therapy (irradiation + TMZ) did not (p = 0.5). Notably, ISCADOR Qu, if used as an adjuvant therapeutic in combination with irradiation and TMZ, further provided benefit in the treatment of experimental GBM.

## 4. Discussion

Many novel therapeutic strategies that aimed to block GBM-associated immunosuppression or to evoke antitumor immune responses failed to prolong GBM patient's survival time until today. In the complementary and anthroposophical medicine mistletoe extracts or even pure ML-1 have been used to treat cancer. Promising results have been observed both in preclinical and in clinical trials [8, 9, 19, 28, 29]. In several studies both* in vitro* and* in vivo* adjuvant mistletoe therapy has been shown to boost the immune system and to induce antitumor activity [29–33]. However, these studies show high variability; in particular, the very old studies lack transparency and a clear description of the mistletoe preparation that was used. Several different mistletoe preparations were used in these studies, providing high, intermediate, or low mistletoe lectin contents or contain additional compounds that are present in extracts but absent in purified or recombinant ML-1. Sometimes it is even not clear which mistletoe preparation was used (for review see [34]). This makes the comparison of data achieved* in vitro* and* in vivo* in animals or even in patients difficult.

We were interested whether ML-1 containing drugs like the fermented extract ISCADOR Qu or pure ML-1 preparations like recombinant ML-1 (Aviscumine) or native ML-1 could be used as adjuvant therapeutics to treat GBM. We were interested whether these drugs might support anti-GBM immune responses and might be feasible therapeutics to enhance the effect of the GBM standard therapy that includes, beside surgical removal of the tumor, irradiation and TMZ-based chemotherapy. We have recently published that ISCADOR Qu and Aviscumine enhanced the NK-cell mediated killing of glioma cells [18]. Here we examined and compare the impact of ISCADOR Qu, Aviscumine, and native ML-1 on T-cell mediated glioma cell killing and the underlying mechanisms.

*In vitro*, ISCADOR Qu as well as Aviscumine and native ML-1 enforced the priming and expansion of tumor-specific T-cells and, at least partially, boosted the T-cell-mediated killing of glioma cells, being the ISCADOR Qu-mediated immunosupporting effects superior to that of Aviscumine or native ML-1 (Figure 1). However, in this context, the immune stimulating effects varied depending on the T cell donor. We suggest several mechanisms the immunostimulating effects of ML-1 containing drugs rely on: treatment of glioma cells with ISCADOR Qu, Aviscumine, or native ML-1 enhanced the expression of multiple proinflammatory genes (Figure 2). The upregulation of IL-6 we observed has been already described [24]. Like IL-1B also the enhanced expression of TNF might induce IL-6 and subsequently reduce brain tumor growth by enhancing macrophage recruitment [35]. Enhanced TNF levels have been found in human whole blood treated with LPS plus recombinant ML-1 [36]. PTGS2/COX-2 is a proinflammatory cytokine which is upregulated in most glioma tissue [37]. Whether the further upregulation of COX-2 by ML-1 containing drugs as observed in the present study will be beneficial has to be discussed since it has been published that COX-1 provides tumor-progressive activity, even in glioma [38]. The downregulation of angiotensin converting enzymes (ACE) fits well to the upregulation of proinflammatory factors since a reduction of ACE mRNA has been described in inflammatory rat models [39]. We found only one proinflammatory gene, HMOX1, being downregulated in glioma cells upon treatment with ISCADOR Qu, Aviscumine, or native ML-1. On the one hand, HMOX1 is a cytoprotective, antioxidant, and anti-inflammatory molecule involved in the regulation of innate immune responses. On the other hand, knockdown of HMOX1 in a mouse model of autoimmune encephalomyelitis led to a persistent activation of antigen-presenting cells and of an enhanced infiltration of Th17 cells into the brain [40]. Th17 cells are a subclass of T-helper-cells that play an important role in the defense against infections. In this context there might be an either beneficial or unfavorable effect of a ML-1-mediated downregulation of HMOX1. To clarify this, further investigations will be necessary.

A second mechanism how ML-1 containing drugs might support anticancer immune responses might be an attenuation of the GBM-associated immunosuppression. We have recently identified TGF-*β* to be downregulated by ISCADOR Qu or pure ML-1s [16]. Besides its promigratory function, TGF-*β* is one of the most immunosuppressive cytokines in GBM (for review see [41]). Local delivery of IL-12, a cytokine we have found to be upregulated, abrogated GBM-induced immunosuppression and finally led to tumor clearance in a syngeneic mouse glioma model [42]. BCL2-L1 ensures T-cell survival, being essential for a functional immune system [43]. Contrarily, in glioma cells the antiapoptotic BCL-family member BCL-2 protects against cell death induced by chemotherapy (for review see [44]).

In the present study, a ML-1-mediated increase in proinflammatory and immunomodulating factors was not only observed in glioma cells* in vitro* but also* in vivo* in ISCADOR Qu treated mice (Figure 4(a)). Upregulation of BLC, CCL24, CCL25, MIP-1*α*, CX3CL1, and CSF3 confirms the hypothesis that ML-1 containing drugs might support immunostimulatory functions and by this also anticancer immune effects. In these mice also enhanced TIMP-1 levels were found. TIMP-1 is involved in the regulation of immune responses by inhibiting matrix metalloproteinases (MMP) that provide pro- and anti-inflammatory function as MMPs can cleave pro-IL-1*β* into its active form, but also can degrade it [45, 46]. Additionally, TIMP-1 acts as cytokine by binding to CD63, thus inducing cell growth and promoting survival of granulocytes and B-cells [47].

Stimulation of T-cell-expansion and -activity might be another mechanism how ML-1 containing drugs provide their immunosupporting function. Elevated T-cell-mediated glioma cell killing was mainly observed if during the T-cell priming and expansion period cocultured glioma cells were pretreated with ISCADOR Qu, Aviscumine, or native ML-1 (Figure 1). However, the T-cell stimulatory effects of ML-1 containing drugs showed a strong variability dependent on the donor the T-cells were derived from and dependent on which ML-1 containing preparation was used. Superior T-cell mediated glioma cell lysis was observed if during the T-cell priming period cocultured glioma cells were treated with ISCADOR Qu (3/3 donors) whilst Aviscumine showed this effect in 2/3 and native ML-1 in only 1/3 donors. The superior immunosupporting effect of ISCADOR Qu might rely on the presence of ML-2 and ML-3 as well as on several minor compounds that are present in the extract but are absent in pure ML-1 preparations. Triterpenes, phenolic acids, phenylpropanoids, and flavonoids, all components of ISCADOR Qu, have been published to modulate immune responses [6, 48]. However, further investigation will be needed to examine which of the additional compound(s) present in ISCADOR Qu might be responsible for the superior immunosupporting effect of the fermented extract.

The supporting function in the context of the expansion and activation of T-cells might be based on the enhanced expression of proinflammatory genes by tumor cells but might also be an effect of the presentation of novel tumor-antigens. Even when using ML-1 concentrations that are far below the IC_50_ determined for LNT-229 cells [16], some glioma cells might die by the treatment leading to the release of cytoplasmic proteins that then serve as tumor-specific neo-antigens, finally boosting the expansion of tumor-specific T-cell clones. It could be implicated that viscotoxins present in ISCADOR Qu might support the presentation of tumor-neo-antigens since they induce cell death by cell membrane permeabilization [49]. Additionally, one could speculate that damage-associated molecular pattern proteins (DAMPs) are released by the treated glioma cells. DAMPs elicit an immune response by activating dendritic cells (DC), promoting the release of proinflammatory factors and by recruiting immune cells that infiltrate the tumor area [50]. In this context it has been published that mistletoe extracts stimulated the maturation of DCs [22, 51]. Finally, in immunocompetent mice serial ISCADOR Qu injection might induce the production of antibodies that either positively or negatively modulate immunoreactivity [52]. However, further experiments will be necessary to clarify the underlying immunosupporting mechanisms of ML containing drugs in more detail.

In addition to the immunostimulating effects resulting from the treatment of glioma cells with ML-1 containing drugs, these drugs also reduced the expression of activity marker proteins, induced cell death, and accordingly reduced anti-glioma activity in T-cells (Figure 3). Other groups also reported a concentration-dependent, ISCADOR Qu-mediated toxicity in PBMCs [53]. The adverse effects of ML-1 based drugs in the context of a direct treatment of immune cells with these agents might be explained by the high expression of the ML-receptor CD75s on immune cells, making them vulnerable to ML-mediated toxicity [12, 54]. In this regard we should mention that the ML concentration we used to treat PBMCs (> 2.4 ng/ml) was much higher than the concentration that will be achieved in the serum of cancer patients [55]. In contrast, low-dose treatment can raise the expression of HLA-DQ and IL-2-receptors in lymphocytes both* in vitro* and* in vivo*. Additionally, cancer patients treated with purified ML-1 presented higher levels of complement factor C3 (which we also found to be upregulated, Figure 2(a)) as well as higher counts of activated immune cells [23, 56].

To assess the therapeutic efficacy of ISCADOR Qu* in vivo*, mice harboring syngeneic orthotopic SMA-560 gliomas were treated with ISCADOR Qu in combination with TMZ and irradiation. We only measured the effect of ISCADOR Qu in glioma mice since ISCADOR Qu was the ML-1 containing drug that provides best immunosupporting activity* in vitro*. No single or double treatment led to a significant prolongation of survival. Similar to ISCADOR Qu monotherapy (Figure 4), irradiation and TMZ elicited only a nonsignificant, small increase in survival time confirming the reported TMZ resistance of SMA-560 cells [57]. In contrast, irradiation, TMZ, and adjuvant applied ISCADOR Qu significantly prolonged the survival of glioma mice, indicating that an adjuvant mistletoe therapy, at least with the fermented extract ISCADOR Qu, provided benefit in the treatment of experimental GBM. Since we have not tested pure ML-1* in vivo* we can only speculate on the* in vivo* anticancer immune effects of Aviscumine or native ML-1. Nevertheless, a clinical impact has been observed for Aviscumine in patients with metastatic melanoma, a highly immunogenic tumor [9, 33] which suggests that an immunosupporting activity exists also for Aviscumine.

## 5. Conclusions

Combined, our data suggest that ML-1 containing drugs improve the T-cell-mediated killing of glioma cells by upregulation of proinflammatory genes in the tumor cells and by systemic upregulation of inflammatory and immunomodulating factors which both enhance the expansion and activity of T-cells. In glioma bearing mice, adjuvant ISCADOR Qu treatment, administered in parallel to irradiation and TMZ chemotherapy, conferred a significant survival benefit. Since adjuvant mistletoe therapy is currently in clinical use for other tumor entities, a mistletoe based therapy of glioma patients seems to be reasonable.

## Data Availability

The datasets generated during and/or analyzed during the current study are available from the corresponding author on reasonable request.
